# A Rare Cause of Acute Abdomen: Diagnosis and Management of Adult Colonic Intussusception

**DOI:** 10.5005/jp-journals-10018-1195

**Published:** 2016-12-01

**Authors:** Mehmet Sertkaya, Arif Emre, Eyüp Mehmet Pircanoglu, Fatih Mehmet Yazar, Murat Tepe, Emrah Cengiz, Ali Isler, Halit Vicdan

**Affiliations:** 1 Department of General Surgery, Sutcu Imam University, Kahramanmaras, Turkey; 2 Department of General Surgery Necip Fazil City Hospital, Kahramanmaras, Turkey; 3 Department of Emergency, Sutcu Imam University, Kahramanmaras Turkey; 4 Department of Radiology Sutcu Imam University, Kahramanmaras, Turkey

**Keywords:** Adult intussusception, Colonic lipoma, Diagnosis, Treatment.

## Abstract

Intussusception in adults is very rarely seen, and this cause acute abdomen. A computed tomography (CT) scan, clinical suspicion, history, and a physical examination are important for the diagnosis. We present two cases of colonic intussusceptions induced by lipoma. The cases had similar locations, diagnoses, and management. Both lipomas were located close to the cecum in the ascending colon, and a right segmental colon resection was performed in both cases. The follow-up of both cases was uneventful. Although benign lesions can cause colonic intussusception, the high incidence of malignancy in colonic lesions should always be considered. Therefore, oncologic surgical procedures should be applied. The definitive diagnosis can be made by histopathology.

**How to cite this article:**

Sertkaya M, Emre A, Pircanoglu EM, Yazar FM, Tepe M, Cengiz E, Isler A, Vicdan H. A Rare cause of Acute Abdomen: Diagnosis and Management of Adult Colonic Intussusception. Euroasian J Hepato-Gastroenterol 2016;6(2):179-182.

## INTRODUCTION

Adult intussusception, a rare condition, is an indication for surgery in adults because of the possibility of malignancy and the risk of ischemia or perforation. Intussusception in adults differs from that in children in terms of clinical presentation, etiology, and incidence, and surgery is generally the best treatment choice. As an untreated adult intussusception bears several potentially serious complications, an accurate preoperative diagnosis is very important. Although intussusception remains difficult to diagnose because of different clinical presentations, a computed tomography (CT) scan is the best tool to diagnose adult intussusception. Intestinal lipomas are infrequent nonepithelial tumors that are usually identified incidentally, or when larger ones lead to symptoms due to obstruction, bleeding, or intussusception. In this study, we report two cases of large bowel lipomas that became symptomatic due to intermittent obstructive episodes, bleeding, and colocolonic intussusception.

## CASE REPORTS

### Case 1

**Fig. 1:**
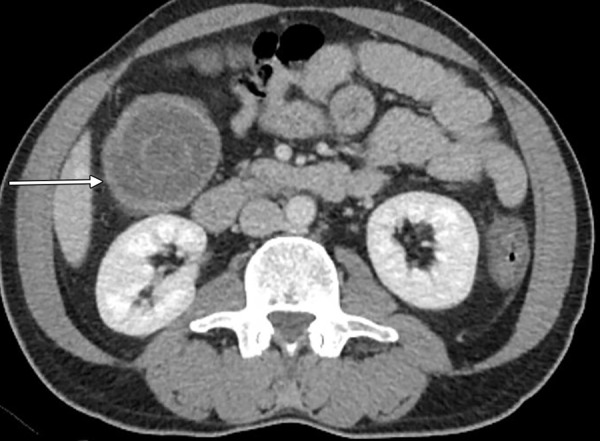
Computed tomography scan of the abdomen

**Fig. 2:**
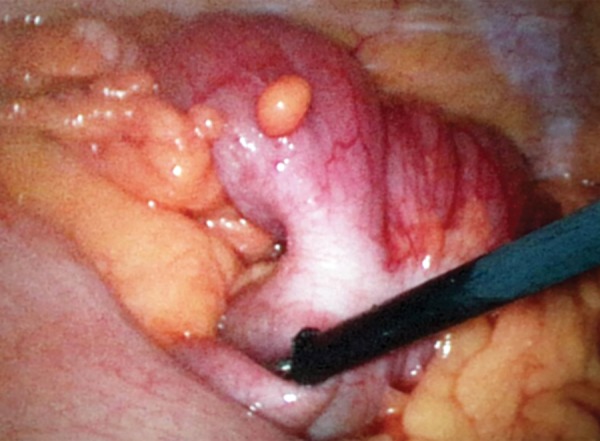
Intussusception of the colon

**Fig. 3:**
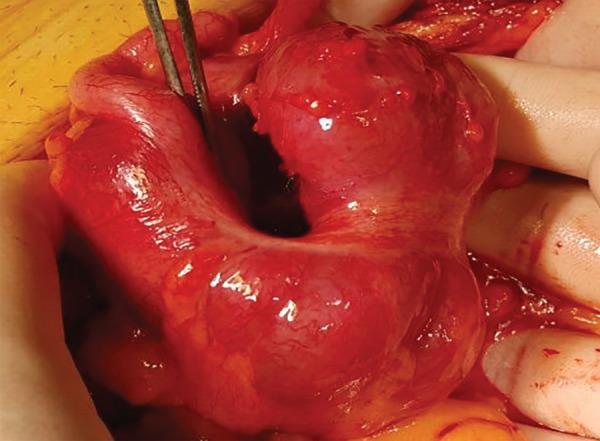
Surgery of the patient

A 50-year-old male was admitted to the emergency service with colicky abdominal pain and nausea without vomiting in the previous day. The patient reported normal defecation. His medical history was unremarkable except for smoking. He had first experienced such pain before 1 month. A physical examination revealed lower-right quadrant tenderness with no defense or rebound, and little stool was found available on a digital rectal examination. Laboratory findings were normal except for leukocytosis. A radiographic examination revealed an air fluid level, but an ultrasonographic examination showed intussusception in 57 mm of the colon, edema in the colon wall, and inflammation in adipose and mesenteric tissues; therefore, a CT scan was performed for further evaluation. The CT scan revealed findings as detected by ultrasonography (Fig. 1). The patient was admitted to the general surgery department, and a laparotomy was performed following a diagnostic laparoscopy. The intussusception was observed in the induced colon (Fig. 2), and the patient was subjected to open surgery (Fig. 3). A segmental right colon resection was performed and the excised tissue was sent for assessment. The findings were benign, so a side-to-side ileocolic anastomosis was performed. The patient had an uneventful recovery, and initiated oral intake on postoperative day 4. The histopathological examination of the specimen revealed a bulging coffee- and maroon-colored lipomatous lesion near the appendix, with a diameter of 4 cm, which was purely benign.

### Case 2

**Fig. 4:**
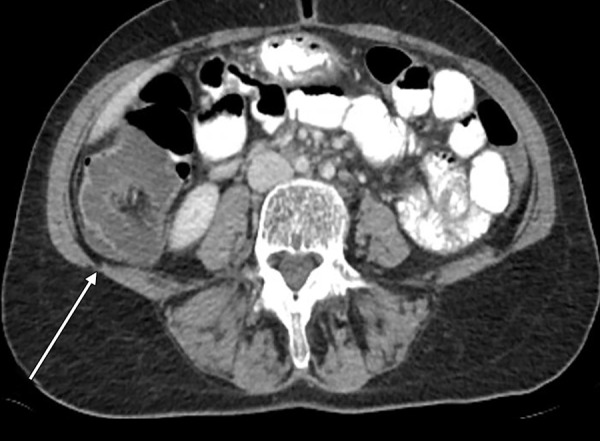
Intussusception of the colon

**Fig. 5:**
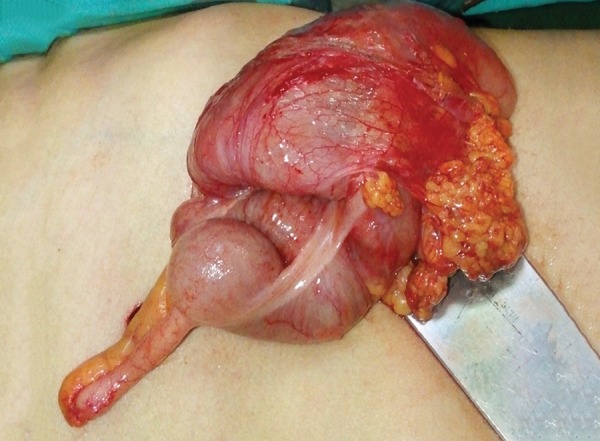
Intussusception of the cecum

**Fig. 6:**
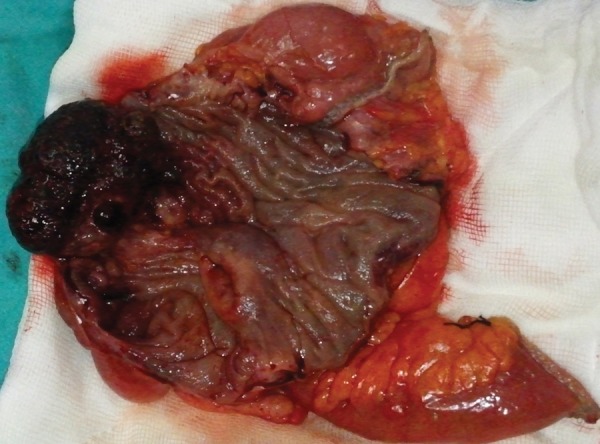
The operated tissue

A 52-year-old woman was admitted to the emergency department with complaints of abdominal pain in the lower-right quadrant, lasting for 3 days, along with nausea and vomiting and an inability to defecate for 1 day. An ultrasonographic examination showed a 56-mm-long invagination of the colon with edema in the lower-right quadrant. An oral contrast radiograph showed an intussusception in the right quadrant; therefore, a CT scan was performed, which revealed an intussusception of the bowel wall in adjacent colonic segments (pneumo intestinalis), suggesting necrosis with minimal free fluid at that level (Fig. 4). Blood tests showed anemia, leukocytosis, and a small increase in C-reactive protein level; other biochemical parameters were normal. A fecal occult blood test was positive, and urinalysis revealed erythrocytes, leukocytes, and bacteria. The patient had a history of mitral valve repair and mitral valve replacement, 25 and 3 years ago respectively, and was taking Coumadin. The patient was hospitalized in the intensive care unit (ICU) and prepared for surgery. A diagnostic laparoscopy was performed, which was proceeded to open surgery due to an intussusception-like image in the cecum (Fig. 5). Following a segmental right colon resection, a side-to-side ileocolic anastomosis was implemented. The excised tissue was sent for evaluation and was reported to be benign. The patient was taken to the ICU postoperatively and defecated smoothly on postoperative day 4. She was discharged from hospital day 7. The histopathological examination of the specimen revealed a dark brown and black-colored polypoid lesion, 4.5 cm in diameter, with a 0.5-cm-long peduncle and 3 cm from the ileocecal valve (Fig. 6). This was interpreted as a torsioned, hemorrhagic, and edematous lipoma.

## DISCUSSION

Intussusception is described as prolapse of a proximal bowel segment into the distal segment. This clinical event was first described by Barbette of Amsterdam in 1674 and was termed, by John Hunter in 1789, as “introssuception,” a rare form of bowel obstruction in adults. Sir Jonathan Hutchinson was the first to operate on a child with intussusception, in 1871.^1-3^

Intussusception in adults is found in <1 of 1,300 abdominal surgeries; the child–adult ratio is >20:1, such that it is almost known as a childhood disease.^2^ Intussusceptions in children are idiopathic in 90% of cases and, generally, can be safely reduced, but a casual lesion is detected in 70 to 90% of all adult cases, which cannot be reduced. Only 1 to 5% of bowel obstructions in adults are caused by intussusception, with a leading point being the main reason.^2-5^ A lipoma was the leading point in both of our cases. Colonic lipomas are generally asymptomatic, and symptoms are correlated with their size. Lipomas are incidentally found during a colonoscopy or surgery for other intentions, and they become symptomatic in 75% of patients when they are >4 cm in size.^6^ The lipomas were 4 cm in our first case and 4.5 cm in the second case; both became symptomatic.

Adult intussusception is a very rare entity, and its etiology differs from that in pediatric patients. About 90% of adult intussusceptions emerge in the small or large bowel, and the remaining 10% involve the stomach or a surgically formed stoma.^2^ A small bowel intussusception can be secondary to an intra- or extraluminal lesion, such as an inflammatory lesion, adenomatous polyp, lymphoma, or metastasis. Malignant lesions are responsible for 30% of cases occurring in the small bowel, whereas up to 66% of cases in the large bowel have a malignant etiology.^1,3,4^ Our cases, which were typical for large bowel intussusceptions, were benign.

Adult intussusception shows clinically uncharacteristic symptoms of bowel obstruction; thus, a diagnosis beyond bowel obstruction is rarely made before surgery. Common physical findings and symptoms are abdominal distension, tenderness and, frequently, an abdominal mass associated with colicky pain, nausea, vomiting, a change in bowel habits, constipation, hypoactivity to absent bowel sounds, and rectal bleeding. Pain with vomiting is the most common symptom, with rectal bleeding being the second most commonly reported symptom.^2-4^ In our first case, the main symptom was colicky pain without vomiting, which was experienced 1 month before becoming acute. However, the second case had more symptoms, such as pain, nausea, vomiting, and constipation, and the fecal occult blood test was positive, indicating rectal bleeding (of which the patient was unaware).

The variability of the clinical presentation makes it difficult to diagnose adult intussusception preoperatively; hence, the diagnosis is usually made during surgery.^2-4^ Plain abdominal radiographs are the first diagnostic method because most cases present with a clinical picture of intestinal obstruction. Air fluid levels are not evident because of the slightly open passage.^1,2,5^ An indistinct air fluid level was seen in our first case. Oral contrast radiographs can be helpful for experienced clinicians, as they may reveal some signs of intussusception, as well as the location. In our second case, the oral contrast radiograph showed signs in the lower right quadrant. Ultrasonography is widely used as a secondary diagnostic tool and is useful for diagnosis; however, this procedure requires appropriate interpretation by an experienced radiologist. Several factors, such as obesity and the presence of massive air in the bowel loops, limit the image quality and diagnostic accuracy associated with this method.^1,2,5^ We used CT to confirm the diagnosis and detect lesions, and our radiologist interpreted the intussusceptions in both cases.

Computed tomography seems to be the best diagnostic method for making a preoperative diagnosis of adult intussusception. Computed tomography is the most important and sensitive diagnostic tool in patients who present with nonspecific abdominal pain. The diagnostic accuracy of CT is 58 to 100%.^2-4^ The characteristic CT imaging features include a target-like or sausage-shaped mass with a layering effect, signs of bowel obstruction, and bowel wall edema with loss of the classic three-layered appearance due to impaired mesenteric circulation.^1-4^ Computed tomography was useful in both of our cases to detect imaging signs, such as a smaller ring within a wider ring (like a target), edema of the bowel wall, and a mass appearance caused by intussusception in the lower-right quadrant (Figs 1 and 4). However, a histopathological examination was needed for definitive diagnosis.

Intussusceptions in children are idiopathic in 90% of cases and pneumatic or hydrostatic reduction treatment is successful in 80% of patients. However, reduction is not strongly advised in adults, because almost 90% of cases are secondary to a pathologic condition that serves as a leading point. In addition, 66% of large bowel intussusception cases have a malignant etiology.^2-4^ Therefore, formal resections using proper oncological procedures are suggested for patients with ileocolic, ileocaecal, and colocolic intussusceptions because of the high incidence of underlying bowel malignancy.^1-4^ According to both the patient’s condition and the surgeon’s experience, an open or laparoscopic surgical procedure can be performed.^2^ In both of our cases, we first evaluated the patients laparoscopically and then moved forward to open surgery. We noticed an intussusception slightly distal to the cecum during the laparoscopic evaluation (Fig. 2). Despite our desire to continue laparoscopically, the emergent condition of the patients restricted our choice. A segmental right colon resection and a side-to-side anastomosis were performed in both cases, although it has been said that the intussusception should be reduced before surgery, to reduce the amount of bowel to be resected. Most authors strongly recommend directly resecting a colonic intussusception because of the high incidence of malignancy.^5^ The resection must be done because reducing a colonic or ileal intussusception due to the risk of recurrence should not be considered sufficient treatment. A direct resection seems to be the most reasonable approach, due to the high risk of malignancy, as well as to prevent the spread of colonic lesions that lead to intussusception.^5^

## CONCLUSION

Intussusception is rare in adults, and an intussusception bound to a colonic lipoma is also seen less frequently. Although CT is the most important diagnostic tool to detect an intussusception, a histopathological evaluation is needed to confirm a benign or malignant lesion. Because intussusceptions in adults usually depend on the presence of a lesion, colonic lesions have a high probability of malignancy and should be resected.
